# Views of physiotherapists on factors that play a role in ethical decision-making: an international online survey study

**DOI:** 10.1186/s40945-022-00157-y

**Published:** 2023-02-01

**Authors:** Andrea Sturm, Roswith Roth, Amanda Louise Ager

**Affiliations:** 1 Interuniversity College for Health and Development Graz, Castle of Seggau, Seggauberg 1, A-8430 Leibnitz, Austria; 2 University of Graz, Institute of Psychology, Universitaetsplatz 2, 8010 Graz, Austria; 3 Doctoral School of Life Sciences and Medicine, Gent University, Campus UZ Gent, Corneel Heymanslaan 10, B3, ingang 46, 9000 Ghent, Belgium

**Keywords:** Ethical decision-making, Code of ethics, Framework, Physiotherapy, World Physiotherapy regions, Knowledge translation, Ethics education

## Abstract

**Background:**

There is a lack of knowledge about the ways physiotherapists around the world learn about professional code of ethics and ethical decision-making frameworks. The profession has a gap in the understanding about physiotherapists’ views on factors that play a role in ethical decision-making and whether these views differ between World Physiotherapy regions.

**Methods:**

An online survey study in English was conducted from October 2018 to October 2019. Participants included 559 physiotherapists located in 72 countries. The self-designed survey questionnaire contained 13 items asking about demographic information and means of learning about ethical codes and decision-making frameworks. A further 30 items were presented which included statements underpinned with individual, organisational, situational and societal factors influencing ethical decision-making. Participants were asked to express their level of agreement or disagreement using a 5-point-Likert-scale.

**Results:**

Participants’ highest rated responses endorsed that the professional role of physiotherapists is linked to social expectations of ethical behaviour and that ethical decision-making requires more skills than simply following a code of ethics. A recognisable organisational ethical culture was rated as supporting good ethical decisions. Comparing responses by World Physiotherapy regions showed significant differences in factors such as culture, religion, emotions, organisational values, significant others, consequences of professional misconduct and professional obligations. Entry level education was not perceived to provide a solid base for ethical decision-making in every World Physiotherapy region. Participants reported multiple sources for learning about a professional code of ethics and ethical decision-making frameworks. What’s more, the number of sources differed between World Physiotherapy regions.

**Conclusions:**

Multiple factors play a role in physiotherapists’ ethical decision-making internationally. Physiotherapists’ ethical knowledge is informed by, and acquired from, several learning sources, which differ in both quality and quantity amongst World Physiotherapy regions. Easily accessible knowledge and education about professional codes of ethics and ethical decision-making can foster continuing professional development for physiotherapists. The establishment of constructive ethical cultures in workplaces can improve ethical decision-making, and should acknowledge the influence of individual, organisational, situational and societal factors. The establishment of collaborative learning environments can support knowledge translation which acknowledges practice-based methods of knowing and learning.

**Supplementary Information:**

The online version contains supplementary material available at 10.1186/s40945-022-00157-y.

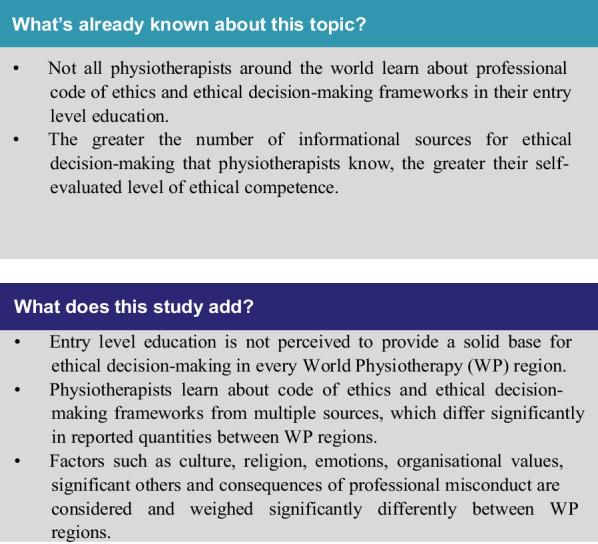



## Introduction

Societal and cultural systems have been identified as key influences on physiotherapists’ ethical practice internationally [1, 2]. Ethical practice is a domain of physiotherapist practice competence, which the World Physiotherapy Association expects graduates to have after completion of the entry-level programme [3]. The educational content of entry-level programmes worldwide may differ depending on a country’s basic conditions for physiotherapists to work either autonomously as first contact practitioners or based on referrals from other health professionals [4, 5], the permitted scope of practice, features of healthcare systems (e.g. private/privatised, public, or fragile systems in conflicting areas) [6–8] or cultural dimensions [9–12]. The minimum qualification required to practice varies greatly between countries and ranges from Diploma (e.g. Germany, Japan, Niger), Bachelors (e.g. Brazil, Australia, Sweden), Masters (e.g. Canada, Ukraine, Mali) to Professional Doctorate (United States, Puerto Rico) [4]. Not all practicing physiotherapists internationally learned about a code of ethics and ethical decision-making frameworks during their entry-level education [2]. Learning formally about a code of ethics has been described as a predicting factor for experiencing ethical situations less frequently by physiotherapists [2]. Also, a relationship of formal (ethics) education and the development of moral judgement has been identified [13–16], but is not generalizable to all regions, populations or professions [17–19]. The adequacy and location of entry-level education were further identified to be risks to competence [20, 21]. Professional learning is not constrained to structured classroom-settings, but can be understood as a social phenomenon [22]. Learning from experience, colleagues, mentoring, tutoring or internet research have been identified as informal learning activities; contributing to highly individualised experiences from a variety of knowledge sources [22–24].

It has been noted by several authors that when it comes to ethical decision-making, physiotherapists require the ability to sensitively recognise an ethical situation [8, 25–27]. They need to holistically understand ethical issues and moral values at stake, as well as to know the ethical principles, theories, professional obligations and the national rules that govern the scope of their practice. Before making a decision and determining and implementing a plan of action, it is important to gather and discuss all relevant information with others involved in the ethical decision-making process [8, 28–30]. Social, cultural and/or religious values and behaviours that influence decisions within health care settings can differ greatly between World Physiotherapy regions [31–34]. There is also great variability in the understanding of the body, health, illness and healing [35, 36] and work conditions [6, 27, 37] around the world. Moral, professional, religious or political theories held by individual clinicians can conflict with particular ethical principles [38], as well as the values and goals of physiotherapists with those of other professionals [29], their work systems [1] and their patients [39]. Physiotherapists’ values – both personal and professional – can dictate behaviours, influence actions, and reflect their attitudes [40]. Feelings and emotions, such as uncertainty, anger and frustration or gratitude, happiness and compassion [41–43] also affect decisions of health care professionals [43–45], but the experience and expression of emotions varies depending on physical and social environments [46]. Furthermore, an overwhelming influence of context has been reported as contributing to ethically challenging situations experienced by physiotherapists globally, ranging from individual professional misconduct, to unethical workplace cultures and even political threats [1].

Ethical decision-making frameworks can support the identification of ethical issues, as well as structure and guide the complex and individual reasoning processes that promote ethically informed clinical decisions [8, 47, 48]. In the light of a shifting focus from professional conduct and the therapeutic relationship to the wider contextual and societal dimensions of physiotherapists’ ethical practice [49], frameworks for ethical decision-making have been introduced that moved away from principlism [50] and embrace the multi-dimensionality of decision-making processes and interrelatedness of protagonists involved. For example, the components of the Realm–Individual Process–Situation (RIPS) Model of Ethical Decision-Making support the identification of elements of ethical situations within individual, organisational/institutional and societal realms. They help to evaluate the required processes of moral reasoning (moral sensitivity, moral judgment, moral motivation, moral courage) [51] as well as the type of ethical situation confronted with (issue or problem, dilemma, distress, temptation, silence) [8]. To address injustice and inequity – whilst acknowledging different sources of knowledge that inform decisions of both physiotherapists (normative ethics) and their patients/clients (narrative or relational ethics) – the Ethical Reasoning Bridge (ER-Bridge) fosters reflection on ethical situations, mutual understanding and the realisation of the capacity to act morally for a change [48, 52]. A phenomenological approach to ethical decision-making, which includes components such as critical thinking and mindful and reflexive practices of physiotherapists, facilitates the interpretation of the individual experience and the narrative understanding of illness by the patient/client [38], whilst acknowledging human connection and interrelatedness. The more sources for informing ethical decision-making are known by physiotherapists, the higher they self-evaluate their level of ethical competence [25].

Within the physiotherapy literature, a lack of knowledge about factors that influence ethical decision-making has been recognised [53]. Indeed, specific factors that play a role in these complex processes have been explored by disciplines such as business and management ethics research for nearly four decades [54–59]. The aggregate of the international literature to date about professional ethics and ethical decision-making, highlights diverse aspects that are important to consider, but does not explore specific factors on individual, organisational, situational and societal levels, which may influence ethical decision-making of physiotherapists. Thus, the objective of the study is to explore how physiotherapists perceptually weigh statements that include different individual, organisational, situational and societal factors that play a role in ethical decision-making; with an aim to probe the relevance of these factors for the physiotherapy profession, as well as to identify and describe differences in perceptual weighing between World Physiotherapy regions [60]. Furthermore, we intend to investigate ways in which physiotherapists learn about code of ethics and ethical decision-making frameworks, ultimately informing their professional practice in international contexts.

## Methods

### Design

A self-designed questionnaire was used to explore the perceptual weighing of various statements related to factors in ethical decision-making amongst physiotherapists, and to ask if, and by which means, participants learned about professional ethics. The overall thematic categorisation of factors in ethical decision-making – individual, organisational, situational and societal – was adopted from business and management ethics [54–56, 58, 59], as well as 12 individual factors and 15 organisational, situational and societal factors identified as playing a role in ethical decision-making processes (Additional file 1: Appendix 1). Physiotherapy literature on ethical decision-making was referred to for supporting and justifying the use of the chosen, predetermined factors and their thematic categorisations [8, 26, 27, 38, 43, 48, 50] (Additional file 1: Appendix 1). The questionnaire was developed in English, and piloted by eight physiotherapists located in Europe, Asia and Australia; six of which English was not their native language. Based on their feedback, the possibility to use a dictionary in case of uncertainty was included into the survey introduction, some amendments of response-options and survey-wording were made, and explanations for question-contexts were added. Ethical decision-making was explained and defined to participants as follows:

“Ethical decision-making helps physiotherapists to make difficult choices when faced with an ethical situation. An ethical situation can be any issue in which an ethical tension is created in the physiotherapist’s practice — for example, a conflict of values, beliefs, or norms; uncertainty as to the appropriate ethical action to take, or distress arising from an inability to act in a way that meets the professional’s (or the profession’s) ethical standards. Ethical decision-making includes recognising the ethical situation, making professional ethical judgements, establishing a moral intent and implementing ethical actions/engaging in ethical behaviour” [8].

The final online survey was shared with physiotherapists internationally using the SurveyMonkey© tool (Version April 2018) between October 2018 and October 2019. The survey started with information about the study, outlining its voluntary and anonymous nature, and asked participants for their voluntary and informed consent. Participants could respond to as many questions as they wished, and leave the survey at any point. The study was ethically approved by the Institute for Ethics and Right in Medicine of the University of Vienna (Vote number 3/2018).

The survey itself consisted of 44 questions within three sections: Section I contained 13 items concerning sociodemographic information (age, gender, religion, nationality) and vocational and education variables (level of physiotherapy education, type of workplace, country and area of workplace, years and fields of physiotherapy practice). Two items asked participants if, and by which means, they learned about codes of ethics and ethical decision-making frameworks. Section II of the survey contained 30 items asking participants about their perceptual weighing of statements including, or underpinned with, various factors which may play a role in their ethical decision-making (Additional file 1: Appendix 1). Participants could indicate how much they agreed or disagreed with the statements using a 5-point-Likert-scale [61]. This section was further divided into two main themes: (A) Individual factors related to the physiotherapist (16 items) and (B) Situational, organisational and societal factors (14 items). Themes and items were presented in a randomised order. Section III provided an optional opportunity to add any other factor(s) playing a role in the participant’s ethical decision-making that they felt were not covered within the previous statements in section II, in order to identify a wider scope of factors than those predetermined. The results from section I and II are presented in this paper. The results from section III are presented in another paper [62].

### Participants and data collection

Physiotherapists from all World Physiotherapy (WP) regions were invited to participate, who had internet-access and a functional understanding of English to complete the questionnaire. The survey was distributed online using purposeful and snowball sampling within professional networks by contacting national physiotherapy associations, promoting the survey with a paid advertisement on the free online database Physiopedia, the distribution of printed hand-outs by AS at the World Physiotherapy (WP, former WCPT) Conference in Geneva 2019 as well as through social media (Facebook, Twitter). The survey was available between October 2018 and October 2019. Five hundred fifty-nine participants from 72 countries completed section I of the survey. Four participants were excluded as their responses were unrelated to physiotherapy. Eight participants self-identified as physiotherapy students. Four hundred fifteen participants completed the survey in its entirety (sections I and II).

### Data analysis

Data was exported from SurveyMonkey. Quantitative data was analysed using the Statistical Package for the Social Sciences software (SPSS version 27.0, IBM Corp., Armonk, NY, USA) by RR. Participants’ sociodemographic characteristics were analysed by Chi^2^, analyses of variance, comparisons of means and descriptively by percentage. Not all participants answered all items; therefore, within the tables the respective numbers of respondents for each item are presented. In a drop-out analysis the sociodemographic and occupational differences between participants who completed the questionnaire and those who dropped out earlier were evaluated.

Participants’ responses to the Likert scale were converted to numbers, using the values 1 = strongly disagree; 2 = disagree; 3 = neither agree nor disagree; 4 = agree; 5 = strongly agree; and treated as intervall data [61]. Means (M) and standard deviations (± SD) for each item were calculated. Comparisons of means were used to identify differences between participants’ responses from different WP regions within the items 14-43, as parametric tests are recognised to produce robust results even with small sample sizes or data that are not normally distributed [63]. Additionally, three groups of percentage ratios of participants’ responses were calculated for each item and WP region: agree (responses to “agree” and “strongly agree”), neutral (responses to “neither agree nor disagree”) and disagree (responses to “strongly disagree” and “disagree”). We provided a ranking order of the means of the statements in order to clarify participants’ perceptual weighing. The five WP regions are understood to be Africa (AR), Asia Western Pacific (AWPR), Europe (ER), North America Caribbean (NACR) and South America (SAR).

## Results

### Participant demographics

Five hundred fifty-five individuals in the age range of 18 to 81 years (Table 1) participated in the online survey. 379 (68.4%) participants were female, 170 (30.7%) male and five (0.9%) diverse (one participant did not indicate their gender), located across 72 countries. Table 1 presents the distribution of age (F_2,550_ = 0.915, *P* = .401), WP region (Chi^2^=6.63, *P* = .576) and religion (Chi^2^=13.10, *P* = .041) by gender. We designated four groups of religions. Most of the participants (45.2%) belonged to Christianity; 23.6% described themselves as non-religious; 22.4% avow to be Muslims, Buddhist and Hinduists; and 9.1% follow other religions or did not want to share this information. More females and less males than expected were Christians, and more males and less females than expected confessed to be non-religious; two female participants did not answer this question.

**Table 1:** Sample description: age, nationality (geographic/WP region), and religion by gender

	N (%) or M (±SD)	N (%) or M (±SD)	N (%) or M (±SD)	N (%) M (±SD)
	Female	Male	Diverse	Total
**Gender**	379 (68.4%)	170 (30.7%)	5 (0.9%)	554 (100%)
**Age yrs. M (±SD)**	37.38 (±11.86)	36.24 (±11.88)	32.40 (±8.56)	36.98 (±11.84)
**Geographic region (WP member + non-member countries)**				
**African region (AR)**	42 (7.6%)	14 (2.5%)	1 (0.2%)	57 (10.2%)
**Asia Western Pacific region (AWPR)**	100 (18.0%)	50 (9.0%)	1 (0.2%)	151 (27.2%)
**Europe region (ER)**	186 (33.6%)	85 (15.4%)	1 (0.2%)	273 (49.2%)
**North America Caribbean Region (NACR)**	43 (7.8%)	18 (3.2%)	2 (4.3%)	63 (11.4%)
**South America Region (SAR)**	8 (1.5%)	3 (0.5%)	0 (0%)	11 (2.0%)
**Religion**				
**Christianity**	186 (33.7%)	61 (11.1%)	2 (0.4%)	249 (45.2%)
**Islam, Hinduism, Buddhism**	82 (14.8%)	42 (7.6%)	0 (0%)	124 (22.4%)
**Secular/non-religious/agnostic/atheist/irreligious/unaffiliated**	76 (13.8%)	52 (9.4%)	2 (0.4%)	130 (23.6%)
**Ethnic and indigenous religion, Sikhism, Juche, Spiritism, Judaism, Baha’i, Jainism, Neo-Paganism, Unitarian, Don’t want to share, Other**	33 (6.2%)	15 (2.8%)	1 (0.2%)	49 (9.1%)

Table 2 shows the main vocational characteristics of the participants by gender. Participants worked on average for 12.90 years as physiotherapists (F_2,550_ = 0.600, *P* = .549), reported 2.59 different types of workplaces currently and/or throughout their career (F_2,550_ = 0.600, *P* = .549), and were practicing in 4.90 different physiotherapy fields (F_2,551_ = 0.507, *P* = .603). Most participants worked in urban (73.8%) or urban and rural (16.7%) areas, fewest in rural areas only (9.5%), no statistically significant gender differences were found.

**Table 2:** Sample description: vocational variables by gender

	N (%) or M (±SD)	N (%) or M (±SD)	N (%) or M (±SD)	N (%) or M (±SD)
	Female	Male	Diverse	Total
**Years in work (without students,** ***N*** **= 539)**				
**Working yrs. M (±SD)**	13.38 (±11.40)	12.03 (±11.18)	5.00 (±4.40)	12.90 (±11.33)
**Type of workplace**				
**Private**	382 (69.07%)	163 (29.48%)	3 (0.54%)	548 (99.09%)
**Government/public**	291 (52.62%)	86 (15.55%)	4 (0.72%)	381 (68.89%)
**Teaching institution**	81 (14.65%)	46 (8.32%)	1 (0.18%)	128 (23.15%)
**Research institution**	16 (2.89%)	15 (2.71%)	1 (0.18%)	32 (5.79%)
**Sports club**	21 (3.80%)	20 (3.62%)	2 (0.26%)	43 (7.78%)
**Self-employed/owner**	176 (31.83%)	87 (15.73%)	1 (0.18%)	264 (47.74%)
**Other**	36 (6.51%)	7 (1.27%)	0 (0%)	43 (7.78%)
**Average of workplaces currently or over career**				
**Workplaces M (±SD)**	2.64 (±1.53)	2.49 (±1.53)	2.40 (±0.89)	2.59 (±1.52)
**Working area**				
**Rural area**	29 (5.5%)	20 (3.8%)	1 (0.2%)	50 (9.5%)
**Urban area**	273 (51.8%)	113 (21.4%)	3 (0.6%)	389 (73.8%)
**Both areas**	56 (10.6%)	31 (5.9%)	1 (0.2%)	88 (16.7%)
**Different fields of work (including students)**				
**Acupuncture/dry needling**	52 (9.39%)	36 (6.50%)	0 (0%)	88 (15.9%)
**Animal practice**	2 (0.36%)	2 (0.36%)	0 (0%)	4 (0.7%)
**Aquatic therapy**	67 (12.09%)	11 (1.99%)	0 (0%)	78 (14.1%)
**Cardiorespiratory**	124 (22.38%)	47 (8.48%)	3 (0.54%)	174 (31.4%)
**Disability (intellectual and physical)**	83 (14.98%)	36 (6.50%)	2 (0.36%)	121 (21.8%)
**Education in physiotherapy**	105 (18.95%)	51 (9.21%)	0 (0%)	156 (28.1%)
**Health promotion**	83 (14.98%)	40 (7.22%)	2 (0.36%)	125 (22.5%)
**Information management/ administration**	33 (5.96%)	13 (2.35%)	1 (0.18%)	47 (8.5%)
**Mental health**	31 (5.60%)	8 (1.44%)	1 (0.18%)	40 (7.2%)
**Neurology**	144 (25.99%)	64 (11.55%)	2 (0.36%)	210 (36.8%)
**Occupational health and ergonomics**	44 (7.94%)	27 (4.87%)	0 (%)	71 (12.8%)
**Oncology/palliative care**	64 (11.55%)	16 (2.89%)	0 (%)	80 (14.4%)
**Orthopedics/manual therapy**	246 (44.40%)	118 (21.30%)	2 (0.36%)	366 (65.9%)
**Older people**	161 (29.06%)	62 (11.19%)	0 (0%)	223 (40.2%)
**Pediatrics**	115 (20.76%)	30 (5.42%)	0 (0%)	145 (26.1%)
**Rehabilitations**	236 (42.60%)	105 (18.95%)	2 (0.36%)	343 (61.8%)
**Research**	55 (9.93%)	30 (5.42%)	2 (0.36%)	87 (15.7%)
**Sport physiotherapy**	115 (20.76%)	92 (16.61%)	1 (0.18%)	208 (37.5%)
**Women’s, men’s and pelvic health**	82 (13.00%)	18 (3.25%)	0 (%)	100 (18.0%)
**Other**	40 (7.22%)	10 (1.81%)	1 (0.18%)	51 (9.2%)
**Average of different working fields**				
**Working fields M (±SD)**	4.97 (±3.41)	5.05 (±3.10)	3.80 (±3.39)	4.90 (±3.04)

Tables 3 presents the highest degree achieved in physiotherapy or other disciplines, and the means and sources of learning about code of ethics and ethical decision-making frameworks by gender. Some participants indicated more degrees, on average 1.16; there were no degree differences observed between gender (F_2,550_ = 2.887, *P* = .057) and WP regions (F_4,549_ = 1.298, *P* = .270).

**Table 3:** Sample description: highest academic degree (in physiotherapy and other disciplines), and sources of learning about codes of ethics and ethical decision-making frameworks by gender

	N or M (±SD)	N or M (±SD)	N or M (±SD)	N (%) or M (±SD)
	Female	Male	Diverse	Total
**Highest level of educational degree achieved (in physiotherapy or other discipline)**				
**Bachelor’s degree/diploma**	200 (36.17%)	78 (14.10%)	3 (0.54%)	281 (50.81%)
**Graduate diploma**	50 (9.04%)	26 (4.70%)	1 (0.18%)	77 (13.92%)
**Master’s degree**	115 (20.79%)	69 (12.47%)	1 (0.18%)	185 (33.85%)
**Professional doctorate**	32 (5.79%)	12 (2.17%)	1 (0.18%)	45 (8.14%)
**Research doctorate**	12 (2.17%)	5 (0.90%)	1 (0.18%	19 (3.44%)
**Other**	25 (4.52%)	10 (1.81%)	0 (0%)	35 (6.33%)
**Average academic degrees**				
**Academic degrees M (±SD)**	1.15 (±0.41)	1.18 (±0.44)	1.60 (±1.34)	1.16 (±0.43)
**Learning about a professional code of conduct or a code of ethics for physiotherapists**				
**No**	57 (10.29%)	22 (3.97%)	1 (0.18%)	80 (14.4%)
**Yes, during my basic physiotherapy education**	226 (40.79%)	100 (18.05%)	2 (0.36%)	328 (59.1%)
**Yes, in a graduate or post-graduate program**	74 (13.36%)	35 (6.32%)	0 (0%)	109 (19.6%)
**Yes, in a professional ethics course**	39 (7.04%)	16 (2.89%)	1 (0.18%)	56 (10.1%)
**Yes, by learning about professional ethics on my own**	72 (13.00%)	39 (7.04%)	0 (0%)	111 (20.0%)
**Yes, by learning about professional ethics from others**	46 (8.30%)	22 (3.97%)	0 (0%)	68 (12.3%)
**Don’t know**	15 (2.71%)	6 (1.08%)	1 (0.18%)	22 (4.0%)
**Other**^a^	13 (2.35%)	5 (0.90%)	0 (0%)	18 (3.2%)
**Average of learning sources about a professional code of conduct or code of ethics for physiotherapists**				
**Learning Sources M (±SD)**	1.27 (±0.96)	1.30 (±0.98)	0.80 (±0.45)	1.27 (±0.96)
**Learning about specific ethical decision-making or ethical reasoning frameworks**				
**No**	100 (18.08%)	41 (7.41%)	1 (0.18%)	142 (25.6%)
**Yes, during my basic physiotherapy education**	169 (30.56%)	83 (15.01%)	1 (0.18%)	253 (45.6%)
**Yes, in a graduate or post-graduate program**	70 (12.66%)	36 (6.51%)	1 (0.18%)	107 (19.3%)
**Yes, in a professional ethics course**	37 (6.69%)	17 (3.07%)	1 (0.18%)	55 (9.9%)
**Yes, by learning about professional ethics on my own**	70 (12.66%)	36 (6.51%)	3 (0.54%)	109 (19.6%)
**Yes, by learning about professional ethics from others**	54 (9.76%)	21 (9.22%)	2 (0.36%)	77 (13.9%)
**Don’t know**	23 (4.16%)	12 (2.17%)	0 (0%)	35 (6.3%)
**Other**^b^	11 (1.99%)	2 (0.36%)	0 (0%)	13 (2.3%)
**Average of learning sources about specific ethical decision-making or ethical reasoning frameworks**				
**Learning sources M (±SD)**	1.12 (±1.03)	1.21 (±1.04)	1.60 (±1.14)	1.15 (±1.03)

^a^National Professional Associations on course of professional registration or as a provider of ethics resources; as part of participants’ individual professional role such as teaching students in professional ethics, being part of a Professional Council Ethics Committee, or assessing physiotherapy degree courses; as part employment contracts containing organisational codes of conduct; on course of required continued professional development (CPD); national standards for health professionals such as the British ‘Duty of Candour’, a professional obligation of healthcare professionals to tell the truth to patients when a procedure fails

^b^Information provided by lawyers specialised in ethics; learning from practical experience; CPD courses (described as providing information about areas that participants would otherwise have not been exposed to, or remaining unknown); as an effect of applying for ethical clearance for research projects; readings related to specific diagnoses; trainings required for specific work settings such as palliative care or hospice; personal religious convictions and moral values

The comparisons between the WP regions showed significant differences in the number of sources for learning about code of ethics (F_4,550_ = 4.223, *P* = .002) as well as in learning about ethical decision-making frameworks (F_4,549_ = 4.035, *P* = .003) between the ER and the NACR (see Table 4). For the ER were the fewest, for the NACR were the most learning sources reported.

**Table 4:** Learning sources about professional code of conduct or code of ethics (Q12) and ethical decision making frameworks (Q13) by WP region

Learning sources	N	Item 12 Average M (±SD)	Item 13 Average M (±SD)
**Africa region (AR)**		1.49 (±1.09)	1.30 (±1.13)
**Asia Western Pacific region (AWPR)**		1.26 (±0.88)	1.21 (±1.05)
**Europe region (ER)**		1.15 (±0.90)	1.01 (±0.94)
**North America Caribbean region (NACR)**		1.62 (±1.18)	1.54 (±1.20)
**South America region (SAR)**		1.55 (±0.93)	1.09 (±0.94)
**Total**		1.28 (±0.96)	1.15 (±1.03)

Of the 555 participants, 280 participants (50,45%) absolved their entry-level education in countries, where direct access is fully permitted; 69 participants (12,43%) trained in countries, where direct access is permitted private only; 184 participants (33,16%) trained in countries, where direct access is not permitted; for 22 participants (3,96%) the information was not available.

Of the 66 countries, in which participants absolved their entry-level education, 23 countries (34,85%) fully permit direct access; 21 countries (31,82%) permit direct access only private; 14 countries (21,21%) do not permit direct access; for eight countries (12,12%) the information was not available (Reference year 2021) [4]. As the average of respondents worked for about 13 years as physiotherapists, the situation pertaining to direct access at the time of their entry-level education was probably different in several countries.

### Flow of participants throughout the study

One hundred fifty participants answered the sociodemographic and working-related items but did not complete the survey (25.27% of total). The drop-outs consisted of 94 females, 45 males and 1 diverse (completers vs. drop-outs by gender: Chi^2^=0.247, *P* = .884). The drop-outs were significantly younger than completers (mean age 33.69 vs. 38.10 years, F_1,551_ = 14.89, *P* < .001) and worked for a shorter period of time (years: 10.07 vs. 13.82 years, F_1,538_ = 11.24, *P* = .020, *N* = 133, eight students were not counted). The rate of drop-outs across WP regions differed; with most drop-outs coming from the ER (12.2%) and the AWPR (7.9%), fewer from NACR (2.7%), AR (1.8%) and SAR (0.6%) (Additional file 2: Appendix 2). The rates of drop-outs pertaining to religion were: (1) Christianity: 10.9%, (2) Islam, Hinduism, Buddhism: 7.3%, (3) non-Religious: 4% and (4) the remaining group: 2.7% (Chi^2^=9.53, *P* = .023). From groups 2 and 4 more respondents than expected and from group 3 less individuals than expected dropped out of the survey.

Drop-outs reported having less types of different workplaces, currently and over their career, than completers (2.08 vs. 2.8, F_1,552_ = 22.78, *P* < .001), indicated working in fewer physiotherapy fields (4.4 vs. 5.1, F_1,553_ = 5.82, *P* = .016), and were represented in all different working fields. They dropped out in a proportional relation (Chi^2^=3.53, *P* = .171), in comparison to completers in urban, rural or both areas.

Pertaining to learning sources about a professional code of ethics, completers indicated significant more sources than drop-outs (1.33 vs. 1.11, F_1,553_ = 5.82, *P* = .016). Sources for learning about ethical decision-making frameworks showed differences close to significance (M: 1.20 vs. 1.01, F_1,552_ = 3.78, *P* = .052) of fewer sources in dropouts than in completers.

### Rating and ranking of statements

The means of agreement to the statements that participants rated were ranked from highest agreement to lowest agreement (Table 5). The highest approval rates were related to these three survey statements: item 36 *“Physiotherapists need to behave ethically because it is socially expected as their professional role*”; item 27 *“Ethical decision-making requires more skills than just observing a code of conduct or ethical principles”*; and item 34 *“A recognisable organisational culture of ethical practice can help a physiotherapist to make a good ethical decision”*. The lowest agreement levels were scored for the following three survey statements: item 23 *“Ethical decision-making stresses me out”*; item 33 *“Organisational values have greater influence on my ethical decision-making than my own considerations”*; and item 20 *“My religious beliefs play a role in ethical decision-making”*.

**Table 5:** Items 14-43: Ranking of the responses of the total cohort and WP Regions based on M (±SD), N (population) and % of Agree, Neutral and Disagree Responses

				Total			WP Regions									
Item	Description	N	M (±SD)	Rank			Africa		Asia West Pacific		Europe		North Amer Caribbean		South America	
					N	%	N	%	N	%	N	%	N	%	N	%
					Agree		Agree		Agree		Agree		Agree		Agree	
					Neutral		Neutral		Neutral		Neutral		Neutral		Neutral	
					Disagree		Disagree		Disagree		Disagree		Disagree		Disagree	
36	Physiotherapists need to behave ethically because it is socially expected as their professional role	415	4.26 (±0.74)	1	372	89.6	46	97.9	97	90.7	182	88.8	41	85.4	6	75.0
					32	7.7	1	2.1	4	3.7	20	9.8	6	12.5	1	12.5
					11	2.7	0	0	6	5.6	3	1.5	1	2.1	1	12.5
27	Ethical decision-making requires more skills than just observing a code of conduct or ethical principles	450	4.23 (±0.69)	2	412	91.6	45	91.8	111	93.3	197	90.0	49	92.5	10	100
					30	6.7	4	8.2	6	5.0	18	8.2	2	3.8	0	0
					8	1.8	0	0	2	1.7	4	1.8	2	3.8	0	0
34	A recognisable organisational culture of ethical practice can help a physiotherapist to make a good ethical decision	415	4.19 (±0.75)	3	368	88.7	43	91.5	98	91.6	180	87.8	40	83.3	7	87.5
					36	6.5	3	6.4	5	4.7	23	11.2	4	8.3	1	12.5
					11	2.7	1	2.1	4	3.7	2	1.0	4	8.3	0	0
35	How senior physiotherapists represent their profession influences the attitude of junior physiotherapists in a good or bad manner	415	4.16 (±0.81)	4	355	85.5	41	87.2	96	89.7	167	81.5	43	89.6	8	100
					42	10.1	5	10.6	10	9.3	24	11.7	3	6.3	0	0
					18	4.3	1	2.1	1	0.9	14	6.8	2	4.2	0	0
21	In ethical decision-making I consider my professional obligations	450	4.12 (±0.68)	5	399	88.7	47	95.9	98	82.4	194	88.6	51	96.2	9	90.0
					41	9.1	1	2.0	17	14.3	21	9.6	1	1.9	1	10.0
					10	2.2	1	2.0	4	3.4	4	1.8	1	1.9	0	0
14	I can recognise a professional ethical situation	450	4.07 (±0.58)	6	402	89.3	46	93.9	105	88.2	191	87.2	50	94.3	10	100
					43	9.6	3	6.1	13	10.9	25	11.4	2	3.8	0	0
					5	1.1	0	0	1	0.8	3	1.4	1	1.9	0	0
32	Unethical behaviour increases where it stays unpunished	415	4.05 (±0.94)	7	324	78.3	43	91.5	95	88.8	139	67.8	40	83.3	8	100
					57	13.7	3	6.4	8	7.5	42	20.5	4	8.3	0	0
					33	8.0	1	2.1	4	3.7	24	11.7	4	8.3	0	0
22	In ethical decision-making I consider the viewpoints of all involved persons or/and facilities	450	4.04 (±0.70)	8	397	88.2	45	91.8	107	89.9	193	88.1	44	83.0	8	80.0
					34	7.6	1	2.0	8	6.7	19	8.7	5	9.4	1	10.0
					19	4.2	3	6.1	4	3.4	7	3.2	4	7.5	1	10.0
39	Healthcare system conditions can limit or permit unethical practices	415	3.90 (±0.92)	9	323	77.8	44	93.6	83	77.6	147	71.7	41	85.4	8	100
					50	12.0	1	2.1	14	13.1	32	15.6	3	6.3	0	0
					42	10.1	2	4.3	10	9.3	26	12.7	4	8.3	0	0
15	I can analyse and describe a professional ethical situation	450	3.87 (±0.70)	10	359	79.8	38	77.6	100	84.0	166	75.8	46	86.8	9	90.0
					69	12.4	7	14.3	15	12.6	41	18.7	5	9.4	1	10.0
					22	4.9	4	8.2	4	3.4	12	5.5	2	3.8	0	0
17	I can describe the difference between personal morality and professional ethics	450	3.86 (±0.84)	11	356	79.1	41	83.7	100	84.0	163	74.4	44	83.0	8	80.0
					51	11.3	5	10.2	11	9.2	29	13.2	6	11.3	0	0
					43	9.6	3	6.1	8	6.7	27	12.3	3	5.7	2	20.0
40	Contextual factors* can act as barriers or resources to ethical decision-making (Characteristics unique to a particular situation, e.g., geographical location and setting, economic factors, resource pressures, institutional context, payment mechanisms)	415	3.81 (±0.85)	12	313	75.4	40	85.1	80	74.8	150	73.2	38	79.2	5	62.5
					64	15.4	4	8.5	17	15.9	35	17.1	5	10.4	3	37.5
					38	9.2	3	6.4	10	9.3	20	9.8	5	10.4	0	0
29	My personal values play a role in ethical decision-making	450	3.77 (±0.96)	13	322	71.6	34	69.4	84	70.6	159	72.6	38	71.7	7	70.0
					76	16.9	9	18.4	24	20.2	33	15.1	7	13.2	3	30.0
					52	11.6	6	12.2	11	9.2	27	12.3	8	15.1	0	0
43	Possible consequences of the decision play a role in my ethical decision-making	415	3.76 (±0.92)	14.5	303	73.0	37	78.7	79	73.8	149	72.7	32	66.7	6	75.0
					59	14.2	6	12.8	18	16.8	30	14.6	5	10.4	0	0
					53	12.8	4	8.5	10	9.3	26	12.7	11	22.9	2	25.0
18	I feel competent when I need to make a professional ethical-decision	450	3.76 (±0.82)	14.5	319	70.9	32	65.3	85	71.4	148	67.6	45	84.9	9	90.0
					97	21.6	13	26.5	23	19.3	54	24.7	6	11.3	1	10.0
					34	7.6	4	8.2	11	9.2	17	7.8	2	3.8	0	0
28	I have the ability to deal with uncertainty in an ethical situation	450	3.71 (±0.75)	16	315	70.0	33	67.3	86	72.3	145	66.2	44	83.0	7	70.0
					105	23.3	10	20.4	27	22.7	62	28.3	5	9.4	1	10.0
					30	6.7	6	12.2	6	5.0	12	5.5	4	7.5	2	20.0
16	I know the code of ethics for physiotherapists of the country I work in	449	3.70 (±0.95)	17	324	72.2	38	77.6	93	78.2	142	65.1	44	83.0	7	70.0
					58	12.9	6	12.2	11	9.2	36	16.5	4	7.5	1	10.0
					67	14.9	5	10.2	15	12.6	40	18.3	5	9.4	2	20.0
41	Cultural factors of the country I work in play a role in my ethical decision-making	415	3.60 (±1.03)	18	269	64.8	37	78.7	80	74.8	117	57.1	30	62.5	5	62.5
					70	16.9	7	14.9	13	12.1	41	20.0	8	16.7	1	12.5
					76	18.3	3	6.4	14	13.1	47	22.9	10	20.8	2	25.0
38	Ethical decision-making is more difficult when the factors that contribute to the situation are outside of the realm of my influence	415	3.56 (±0.95)	19	265	63.9	33	70.2	71	66.4	125	61.0	32	66.7	4	50.0
					77	13.9	6	12.8	22	20.6	41	20.0	6	12.5	2	25.0
					73	17.6	8	17.0	14	13.1	39	19.0	10	20.8	2	25.0
31	Bureaucracy tends to make physiotherapists to follow rules and obligations rather than to think independently about ethical decision-making	415	3.49 (±0.97)	20	232	55.9	29	61.7	60	56.1	111	54.1	26	54.2	6	75.0
					110	26.5	10	21.3	31	29.0	52	25.4	16	33.3	1	12.5
					73	17.6	8	17.0	16	15.0	42	20.5	6	12.5	1	12.5
37	When ethical behaviour is compromised regularly by a physiotherapist other colleagues will tend to also behave less ethically	415	3.45 (±1.04)	21	230	55.4	27	57.4	74	69.2	91	44.4	31	64.6	7	87.5
					90	21.7	9	19.1	11	10.3	61	29.8	8	16.7	1	12.5
					95	22.9	11	23.4	22	20.6	53	25.9	9	18.8	0	0
42	The type/kind of an ethical situation plays a role in my ethical decision-making (E.g., an ethical dilemma between competing principles which both seem to be right, a distress of which course of action to take, a temptation of choosing between ‘right’ and ‘wrong’ when you would benefit from doing the wrong thing, remaining silent in a situation which appears to be too difficult to be resolved)	414	3.38 (±0.94)	22.5	233	56.3	23	48.9	63	58.9	113	55.4	30	62.5	4	50.0
					95	22.9	12	25.5	21	19.6	51	25.0	11	22.9	0	0
					86	20.8	12	25.5	23	21.5	40	19.6	7	14.6	4	50.0
30	Contextual factors of the working system play a role in my ethical decision-making (Characteristics unique to a particular situation, e.g., setting, economic factors, competitive behaviour, resource pressures, institutional context)	415	3.38 (±1.00)	22.5	229	55.2	34	72.3	64	59.8	104	50.7	24	50.0	3	37.5
					87	21.0	5	10.6	22	20.6	47	22.9	11	22.9	2	25.0
					99	23.9	8	17.0	21	19.6	54	26.3	13	27.1	3	37.5
24	My gut-feelings play a role in ethical decision-making	450	3.37 (±1.00)	24.5	247	54.9	23	46.9	60	50.4	128	58.4	28	52.8	8	80.0
					105	23.3	15	30.6	26	21.8	51	23.3	13	24.5	0	0
					98	21.8	11	22.4	33	27.7	40	18.3	12	22.6	2	20.0
19	I refer to a professional framework for ethical decision-making	450	3. 37 (±0.99)	24.5	239	53.1	32	65.3	79	66.4	97	44.3	24	45.3	7	70.0
					115	25.6	10	20.4	28	21.8	66	30.1	10	18.9	3	30.0
					96	21.3	7	14.3	14	11.8	56	25.6	19	35.8	0	0
26	My emotions play a role in ethical decision-making	450	2.95 (±1.03)	26	163	36.2	12	24.5	34	28.6	95	43.4	19	35.8	3	30.0
					112	24.9	10	20.4	31	26.1	52	23.7	15	28.3	4	40.0
					175	38.9	27	55.1	54	45.4	72	32.9	19	35.8	3	30.0
25	The ethics education in my basic physiotherapy training provided a solid foundation for ethical decision-making (depending on the country this could be Bachelor’s degree, Diploma, Physiotherapy school, in some countries the profession’s entry level is Master’s degree or Professional doctorate)	450	2.90 (±1.14)	27	167	37.1	24	49.0	58	48.7	50	22.8	31	58.5	4	40.0
					101	22.4	9	18.4	26	21.8	55	25.1	9	17.0	2	20.0
					182	40.4	16	32.7	35	29.4	114	52.1	13	24.5	4	40.0
23	Ethical decision-making stresses me out	450	2.77 (±1.00)	28	118	21.3	11	22.4	34	28.6	55	25.1	14	26.4	4	40.0
					132	29.3	11	22.4	37	31.1	64	29.2	16	30.2	4	40.0
					200	44.4	27	55.1	48	40.3	100	45.7	23	43.4	2	20.0
33	Organisational values have greater influence on my ethical decision-making than my own considerations	415	2.71 (±1.04)	29	104	25.1	18	38.3	38	35.5	40	19.5	6	12.5	2	25.0
					106	25.5	12	25.5	30	28.0	52	25.4	11	22.9	1	12.5
					205	49.1	17	36.2	39	36.4	113	55.1	31	64.6	5	62.5
20	My religious beliefs play a role in ethical decision-making	450	2.54 (±1.27)	30	119	26.4	22	44.9	41	34.5	42	19.2	13	24.5	1	10.0
					94	20.9	14	28.6	23	19.3	42	19.2	14	26.4	1	10.0
					237	52.7	13	26.5	55	48.2	135	61.6	26	49.1	8	80.0

The highest level of agreement (“strongly agree” and “agree”; range: 21.3 - 91.6%) by over 80% of the participants was identified for seven items, which also determined the ranking of agreement by the mean values ​​(item 36, 27, 34, 35, 21, 13, 22). Ten items still achieved an agreement by more than 70% of the participants* (item 32, 39, 15, 17, 40, 29, 43, 18, 28, 16). The highest rejection (“strongly disagree” and “disagree”, range 1.1 - 52.7%) by over 40% of the participants occurred with four items (25, 23, 33, 20) and one item (26) was rejected by almost 40% of the participants (38.9%). The other rejections were in the single-digit range from 1.1 to 25% of the participants. The neutral category (“neither disagree nor agree”, range: 6.5 - 29.3%) increased for statements where agreement decreased. It was used by over 20% of the participants for items 18, 28, 31, 37, 42, 30, 24, 19, 26, 25, 23, 33, 20 and indicates that there was some uncertainty when weighing these statements (Table 5).

Only one item showed significant differences in relation to gender (*P* = .015): item 18 “*I feel competent when I need to make a professional ethical decision*”. Male participants felt more competent (M = 3.92, ±SD = .78) than females (M = 3.69, ±SD = .82) and diverse participants (M = 3.60, ±SD = 1.14). Ten items differed in the comparative rankings between WP regions; in six items the ER scored lowest in the neutral category (“neither agree nor disagree” =3) or disagree category (=2). ER participants were not decisive in agreeing to item 35 (M = 3.98, ±SD = .83) asking about a perceived influence of senior physiotherapists’ professional attitude on junior physiotherapists (*P* < .001), in comparison to the AWPR (M = 4.40, ±SD = .74) and SAR (M = 4.38, ±SD = .52); and item 37 (M = 3.23, ±SD = .99) about the influence of regularly compromised ethical behaviour by a physiotherapist on other colleagues (*P* < .001), in comparison to SAR participants (M = 4.00, ±SD = .53), who agreed. The same relationship showed item 32, assuming that unethical behaviour increases where it stays unpunished (*P* < .001); and item 19 exploring the use of a professional ethical decision-making framework (*P* < .001), in which participants from the ER (item 32: M = 3.78, ±SD = .95; item 19: M = 3.19, ±SD = .99) and NACR (item 32: M = 4.00, ±SD = .97; item 19: M = 3.17, ±SD = 1.17) agreed significantly lower than those of the SAR (item 32: M = 4.62, ±SD = .52; item 19 M = 3.90, ±SD = .74). Significant differences between participants from the AR and the AWPR were found with item 21 (*P* = .010), where AR participants’ level of agreement (M = 4.37, ±SD = .64) to consider their professional obligations in ethical decision-making was significantly higher than in the AWPR (M = 4.00, ±SD = .70). AR participants scored higher (M = 3.95, ±SD = .88) in their agreement than ER participants (M = 3.42 ± SD = 1.04) for item 41, asking if cultural aspects play a role in their ethical decision-making (*P* < .001). With regards to item 33, participants from the NACR (M = 2.35, ±SD = .93) disagreed in comparison with those from the AWPR (M = 3.06, ±SD = 1.12) that organisational values have a greater impact on ethical decision-making than their own considerations (*P* < .001). That their emotions play a role in ethical decision-making (item 26) is not agreed (*P* = .013) upon by participants from the AR (M = 2.65, ±SD = .97), but rated neutral by participants located in the ER (M = 3.11, ±SD = 1.01) and SAR (M = 3.10, ±SD = .99). Personal religious beliefs (item 20) do not play a role in ER (M = 2.26, ±SD = 1.20) and SAR participants’ (M = 2.20, ±SD.79) ethical decision-making but are rated neutral (*P* < .001) by AR participants (M = 3.27, ±SD = 1.13). An interesting difference was found in item 25 (*P* < .001): ER participants did not agree that the ethics education in their basic physiotherapy training provided a solid foundation for ethical decision-making (M = 2.55, ±SD = 1.05); they differed significantly from participants located in the NACR (M = 3.36, ±SD = 1.00). This difference was also observed in the average learning sources for ethical codes and ethical decision-making frameworks (Table 4).

## Discussion

Our study investigated ways in which physiotherapists learn about code of ethics and ethical decision-making frameworks in international contexts and their views on factors playing a role in ethical decision-making. Although most participants disagreed with the statement that their entry-level education provided a solid foundation for ethical decision-making, and not all participants learned about both codes of ethics and ethical decision-making frameworks, they did agree on their ability to recognise, analyse and describe ethical situations, to deal with uncertainty and to feel competent in ethical decision-making. These perceived competencies are also reflected in most participants’ disagreement to the notion that ethical decision-making stresses them out, that organisational values have greater impact on their ethical decision-making than personal considerations and that their personal religious beliefs play a role in their ethical decisions. This points to the commitment of physiotherapists to emancipation from influences of individual and organisational factors, and the confidence physiotherapists possess with professional ethical decision-making, despite unfavourable educational or working conditions. Developing and holding up individual moral integrity, especially in the light of organisational pressures, requires courage, as it could sometimes lead to diametrical consequences [1, 62], burn-out or/and result in the decision to leave the job or the profession in general [1, 41, 64, 65]. Participants clearly agreed with the statement that a recognisable organisational culture of ethical practice can help physiotherapists to make good ethical decisions. Therefore, it is about time to question (and act for a change of) those organisational (or systemic) conditions that force physiotherapists to choose either sacrificing themselves for remaining true to their (professional and personal) values or to leave their jobs due to ongoing, unresolvable conflicts of interests that harm their health and moral integrity.

These novel findings also suggest that both successful learning about and practical application of professional ethics and ethical decision-making is not confined to formal education. Complex, lively, iterative and ongoing processes are shaped and informed by many sources and experiences during a person’s career [66], and should be actively supported by easily accessibly ethics knowledge. A recent discussion about the best time for medical ethics teaching recommended an overhaul of the current educational practices [67]. To understand both the ethical nature of a therapeutic relationship, and the provision of health services as a moral enterprise, fundamentals of professionalism and ethical responsibilities must be taught in undergraduate programs [67, 68]. But only clinicians’ practical experiences will help to fully understand ethical dimensions of practice and the application of ethical principles to real and dynamic situations. Strengthening post-qualification education, both formally and informally, for example, by knowledge translation that acknowledges practice-based ways of knowing and learning [22], could be one step to bridge the gap between ethics theory and practice, which has been recognised by physiotherapy scholars for decades [38, 69]. Also, there is a plead for mandatory post-graduate ethics trainings [67], as already required in some countries for physiotherapists’ continuing professional development (CPD) and licensure renewal [70, 71]. As ethical situations emerge on social, cultural, political and organisational levels, a decontextualized understanding of ethics and ethical decision-making must be avoided [1, 54]. Therefore, reflections on and critical discussion of physiotherapists’ practices and experiences, their practice environments and (un)ethical cultures of their workplaces are necessary for professional development. It can further facilitate the uptake and incorporation of ethics research both into ethical decision-making practice, and into national and international physiotherapy associations’ professional ethical codes, guides and frameworks [1, 22, 66]. This can decrease the risk that information contained in guidelines becomes judged as impractical, when not fitting with what is known from a variety of knowledge sources that are informing decisions [22]. Furthermore, collaborative learning environments should be established [22] – both in-person and virtually – and physiotherapists encouraged to share about their lived ethical experiences and knowledges in (public) forum discussions, at professional conferences, and in national physiotherapy journals in order to foster further conversation and understanding [27].

Participants’ perceived abilities to recognise, analyse and describe an ethical situation, as well as their perceived competence in ethical decision-making, are mirrored and supported by other ethics researchers’ works. Naamanka et al. investigated self-evaluated ethical competence of Finnish physiotherapists [25], including knowledge about code of ethics and methods for ethical decision-making. Of their sample, 30% did not know, or poorly knew, their national code of ethics. The majority of physiotherapists consulted colleagues for ethical decision-making, while group discussions were common sources that informed ethical decision-making followed by referring to ethics literature, ethics committees and ethics specialists. To evaluate ethical competence variables such as character strength, willingness to do good, ethical awareness and moral judgement skills were included in their survey. These ethical competence variables can be related to the factors in ethical decision-making ego strength, moral intent(ions), awareness and recognition (of ethical issues), and skills and knowledge that underpinned our survey statements to probe their relevance for the physiotherapy field. Canadian physiotherapists’ ability to recognise an ethical situation was reported by Finch et al. [26]. Although their study found no evidence for the use of a systematic approach for ethical analysis (our study’s participants were rather neutral in weighing the statement about referring to a framework for ethical decision-making), an acknowledgement of gathered information about the context and different protagonists involved, and the influence of peers on ethical decision-making processes were reported. Our results support their findings, which confirm the perceived influence of contextual factors of work systems and environment, healthcare systems’ conditions, significant others and other persons involved in the ethical decision-making process.

One surprising finding from our results was that male physiotherapists perceive themselves as more competent in ethical decision-making than females and diverse; a result contrarily to the study by Naamanka et al. [25], where female physiotherapists were reported as evaluating themselves as ethically more competent than males. One explanation for this finding could be that gender equality in Finland is one of the highest in the world [72], with a tertiary education gross enrolment ratio of females vs. males of 103 to 85 [73]. Even if the majority of the study’s sample was located in Western countries, the largest individual country sample came from India (tertiary education gross enrolment ratio of 15 to 21 [73]). Our study encompassed participants from all WP regions with largely diverse socioeconomic, cultural and political backgrounds, sometimes accompanied with unfavourable indicators of gender equality [74]. Other studies identified male gender as a risk to competence, as well as the country of entry level education, both in scoping review based on rather Western literature [20] and a Canadian study investigating factors that put physiotherapists at risk to not meeting professional standards [21]. Possibly, there is a gender bias in how male, female and gender-diverse physiotherapists self-evaluate their overall ethical decision-making competence. To explore these differences, specific variables that measure ethical competence, as used in the “Physiotherapist’s Ethical Competence Evaluation Tool” (PECET) [25], could be investigated from cross-cultural perspectives.

Other innovative findings include the significant differences between WP regions with the level of agreement to several statements. Significant educational differences between WP regions became apparent when considering the statement about the entry-level education of their country providing a solid basis for ethical decision-making. Participants from the ER disagreed in comparison with those from the NACR. A possible explanation for this finding is that both ethics research and education in physiotherapy have a longstanding tradition in North America [49, 50, 75]. Physiotherapists in the United States (US) and Canada are educated to be autonomous practitioners compared to many European countries, where direct access is either limited to private only (e.g. Norway, Poland or Portugal) or not yet permitted (e.g. Austria, Belgium or Greece) [4]. The first code of ethics for US physiotherapists was introduced in 1935 [49]; more than two decades before the first code of ethics of the World Physiotherapy Association in 1959 [76]. A large proportion of the ER sample originated from German speaking countries, who were reported to have learned significantly less often about both code of ethics and ethical decision-making frameworks in their entry level-education than participants from other European countries [77]. Switzerland and Austria introduced physiotherapy education at academic levels in 2006 [78, 79]. In Germany, the major part of physiotherapy education is provided as a 3-year training at the college level with clinical placements in affiliated hospitals and physiotherapy practices [4, 80], also due to the resistance against the full academisation of a physiotherapy program by the German Medical Chamber [81]. In Germany, a translation of the World Physiotherapy code of ethics was provided in 2017, even though it was a founding member country of the World Physiotherapy Association [76, 82]. Such examples demonstrate how heterogeneous physiotherapy practices and associated professional trainings are globally, while solidifying the need for continued research in this area.

Other significant findings seem to be a reflection of the WP regions’ diverse cultural realities. The AR scored significantly higher than the ER in agreeing to the statement that cultural factors play a role in ethical decision-making. European countries have been identified as mostly culturally homogenous, whereas African and Asian countries were identified as highly culturally diverse [83, 84]. The wider literature reports several dimensions of national cultures [84]. One of these dimensions is the relationship between the individual and the group, described as individualism and collectivism. It is unlikely that any culture will be entirely collectivistic or individualistic; accordingly, any overgeneralisation should be avoided. However, the minority of people in the world live in societies that are identified as being individualistic. African, Asian and Latin American countries were described as largely collectivist [84], where various stakeholders are involved in employment relations, with emphasis on importance of social relationships and networks at workplaces, including respect for authority as a cultural value [85, 86]. Such social networks are regulated by informal and implicit social contracts that are based on trust and commitment. Violations of these rules result in sanctions that are socially enforced even stronger than legal sanctions applied to formal contracts [86]. Most European societies (as well as North American) are considered to be culturally individualistic, with varying degrees of individualism within and among countries. In individualistic countries, ties between people are described as being more loose, with own goals and preferences as the primarily motivators for individual behaviour [84, 87]. In addition, another cross-cultural study observed that people benchmark their own dishonesty depending on the perceived extent of dishonesty in their social environment. Societies with higher material security were identified to be more individualistic and to have less corruption [88].

Such differences could explain why the ER was scoring significantly lower in statements addressing the influences of senior professionals’ behaviour on juniors in comparison with the SAR and AWPR, and negative influences of ongoing misconduct without consequences on other physiotherapists’ behaviour, in comparison with the SAR. Cultural differences between participants from individual and collectivist societies may also have contributed to the significantly lower levels of agreement between the NACR and ER compared to the SAR, to the statements pertaining to the increase of unethical behaviour when it remains unpunished, and to the consideration of a professional framework for ethical decision-making. Although participants from the NACR learned more often, and from a higher number of different sources, about ethical decision-making frameworks than their ER counterparts, both agreed significantly less to this statement than participants from the SAR. Participants from the NACR and the ER were probably socialised to value and pursue independence, resulting in beliefs of personal control, and work settings’ focused on individual actions and autonomy. Therefore, we propose that acknowledging cultural dimensions is important for understanding these findings. A socialisation of participants from the NACR to individualistic values may have also contributed to the disagreement to the statement that organisational values have a greater impact than their own considerations in comparison with participants from the AWPR. More than half of the AWPR’s sample originated from India, with an Individualism Index by Hofstede (IDV) of 48, compared to Australia (IDV 90) or Pakistan (IDV 14)). Another cultural dimension investigated by Hofstede et al. (2010) is the way that countries deal with societal inequalities, measured by the Power Distance Index (PDI). High power distance values were found for most Asian countries, Latin America, African and Arabic speaking countries, leading to increased reliance on work superiors, formal rules and regulations [84].

Participants from the ER disagreed with the statement that their religious beliefs play a role in ethical decision-making, but are rated neutral in participants from the AR. The numbers of persons in Europe who identify themselves as non-religious are higher than in the rest of the world; in some European countries it is up to 70% of the population [89]. Spiritual beliefs of physiotherapists in Nigeria for example, were reported as drivers of physiotherapists’ personal agency when providing frontline services in the early SARS-Cov-2 pandemic, whilst experiencing discrimination and stigma on several levels [90]. Participants from the AR disagreed with the statement that emotions play a role in their ethical decision-making; but it was rated as neutral by participants from the ER and the SAR. In a cross-cultural study, Ghanaian participants considered emotions to be less important to attend to in everyday life; contradictory to their Euro-American counterparts, who gave greater importance to affective experiences [91].

However, cultural dimensions alone are not able to explain all significant differences that we identified in our study. Surprisingly, not just the participants from the ER disagreed with the statement pertaining to the influence of personal religious beliefs on their ethical decisions, but also the participants from the SAR, a region described as being historically heavily influenced by the Catholic church [5]. Although the Catholic church lost some influence more recently due to increased political stability and democratic processes [92, 93], an explanation for this finding is not as apparent as for other items of the survey. This applies as well to the statement about professional obligations which are considered in ethical decision-making, where the AR scored significantly higher than the AWPR, both high culturally diverse and largely collectivistic regions. More detailed research is needed to explore, explain and understand such differences, both in terms of cultural and educational causations and beyond.

### Strengths and limitations

Although the 555 participants in this study were located in 72 different countries from all WP regions, their voices cannot be generalised, nor speak for the global physiotherapy profession, as represented by the World Physiotherapy Association with 685,000 members located in 125 member countries [94]. Only 2% of the survey participants originated from the SAR; therefore, the results are not representative for this entire region. The response rate to our survey can be placed in the middle of other cross-cultural survey-studies, conducted in a comparable period and within the physiotherapy profession; with for example 1307 participants located in 49 countries in a study investigating the use of ultrasound, offered in 20 different languages [95]; or 1212 participants located in 94 countries in a study investigating the type and frequency of ethical situations, offered in the English-language [2]; or 58 participants located in 28 countries in a study investigating perceptions of physiotherapists on Artificial Intelligence, offered in the English language [96]. We acknowledge a potential risk that participants could have responded to some of the survey items in a socially desirable manner as viewed favourably by the profession, therefore creating a response bias or moralistic bias [97–99]. The anonymous nature of the survey should have counterbalanced this potential risk [100]. Additionally, another possible response bias could have been created due to participants with a specific interest in ethics, who were more likely to take part in this study. The online-survey design excluded participants without internet access, contributing to a possible sampling bias; although it may have fostered participation of physiotherapists from regions, who we could have not reached otherwise. The English language of the survey may have excluded interested participants with other linguistic preferences, or could have made it difficult for non-English language speakers to fully understand the survey items. Based on the feedback of non-English native speaking physiotherapists who piloted the study, we included a suggestion to use a dictionary in case of uncertainty in the survey introduction, before giving informed consent to study participation. The physiotherapists who piloted the study were not located in all WP regions, but both in individualistic and collectivistic societies, and countries which either permit or prohibit direct access. As there is a lack of literature concerning factors in ethical decision-making in the physiotherapy profession, factors underpinning the survey statements were adopted from other fields such as business ethics or management ethics (the principal investigator has a previous professional background in the economy before entering the physiotherapy profession). Therefore, possibly other interesting aspects, such as relational dimensions of ethical decision-making, have not been considered and warrant further investigation.

## Conclusions

Ethical decision-making by physiotherapists is influenced by various factors, and informed by knowledge acquired from multiple learning sources, both pre- and post-qualification. Factors that play a role in ethical decision-making investigated by other professional fields are perceived by physiotherapists around the globe as being relevant in their professional ethical decision-making processes, and should be explored in more detail by future studies. Easily accessible ethics education and knowledge will foster continuing professional development, and is recommended to enhance individuals’ ethical competences. The establishment of constructive ethical cultures in the workplace can improve ethical decision-making, and support reflexion and discussion of ethical experiences and decision-making. Incorporating ethics knowledge in everyday practice and learning from lived ethical experiences of physiotherapists within collaborative learning environments and further studies may help to close the gap between ethical theory and practice [1, 22]. Physiotherapists around the globe perceive that ethical decision-making requires more skills than just following a code of ethics, and demonstrate understanding for the high ethical demands of the role of physiotherapists, the influence of significant others, the complexity and context-based nature of ethical decision-making, as well as for the influences of healthcare systems’ conditions, local culture and the nature of ethical issues themselves. There are differences between World Physiotherapy regions in how factors in ethical decision-making are perceptually weighed and individually considered. Cultural and educational aspects may contribute to these differences and warrant further investigation.

## Supplementary Information

**Additional file 1: Appendix 1.** List of survey-items with underpinning or embedded factors in ethical decision-making including literature informing survey development.

**Additional file 2: Appendix 2.** Drop-out analysis by gender, age, nationality (geographic/WP region), and religion.

## Data Availability

The data that support the findings of this study are available on reasonable request from the corresponding author. The data are not publicly available due to ethical restrictions to protect the research participants who provided sensitive information.

## Abbreviations

WP: World Physiotherapy
WCPT: World Confederation for Physiotherapy (re-branded as World Physiotherapy in 2020)
CPD: Continuing Professional Development
AR: Africa region (of World Physiotherapy)
AWPR: Asia Western Pacific region
ER: Europe region
NACR: North America Caribbean region
SAR: South America region
IDV: Individualism Index by Hofstede
PDI: Power Distance Index by Hofstede
