# Supplementary material for: Views of physiotherapists on factors that play a role in ethical decision-making: an international online survey study
Source: Arch Physiother. 2023 Feb 1;13:3. doi: 10.1186/s40945-022-00157-y (PMC9889242; doi:10.1186/s40945-022-00157-y)
Supplement: Supplementary file 1 — Additional file 1: Appendix 1. List of survey-items with underpinning or embedded factors in ethical decision-making including literature informing survey development. [file 40945_2022_157_MOESM1_ESM.docx]

Appendix 1. List of survey-items with underpinning or embedded factors in ethical decision-making including literature informing survey development.

|  | **Section II** | **Underpinning/embedded factors** | **Informing literature** |
| --- | --- | --- | --- |
| **#** | **Items related to individual factors** |  | McDevitt et al., 2007; Swisher et al., 2005; Ferrell and Gresham, 1985 |
| 14 | I can recognise a professional ethical situation | Awareness and recognition (of ethical issues), knowledge, education | Hudon et al., 2015; McDevitt et al., 2007; Finch et al., 2005; Swisher et al., 2005, Jones, 1991; |
| 15 | I can analyse and describe a professional ethical situation | Knowledge, education, skills | McDevitt et al., 2007; Finch et al., 2005; Swisher et al., 2005; |
| 16 | I know the code of ethics for physiotherapists of the country I work in | Knowledge, education, values | Hudon et al., 2015; Greenfield and Jensen, 2010; McDevitt et al., 2007; Swisher et al., 2005; |
| 17 | I can describe the difference between personal morality and professional ethics | Knowledge, education, values | Gillam et al., 2014; Greenfield and Jensen, 2010; McDevitt et al., 2007; |
| 18 | I feel competent when I need to make a professional ethical decision | Skills, ego strength | McDevitt et al., 2007; |
| 19 | I refer to a professional framework for ethical decision-making | Skills, education | Greenfield and Jensen, 2010; McDevitt et al., 2007; Swisher et al., 2005; |
| 20 | My religious beliefs play a role in ethical decision-making | Religion, values | McDevitt et al., 2007; Swisher 2002; |
| 21 | In ethical decision-making I consider my professional obligations | Attitude, education | Hudon et al., 2015; McDevitt et al., 2007; Swisher et al., 2005; Swisher, 2002; |
| 22 | In ethical decision-making I consider the viewpoints of all involved persons or/and facilities | Attitude, education, skills | Hudon et al., 2015; Edwards et al., 2011; McDevitt et al., 2007; Swisher et al., 2005; |
| 23 | Ethical decision-making stresses me out | Skills, feelings/emotions | Greenfield and Jensen, 2010; McDevitt et al., 2007; |
| 24 | My gut-feelings* play a role in ethical decision-making *felt sense of something or someone, a body sensation that is meaningful | Feelings/emotions | Gillam et al., 2014; Greenfield and Jensen, 2010; Gaudine and Thorne, 2007; Swisher et al., 2005; |
| 25 | The ethics education in my basic physiotherapy training provided a solid foundation for ethical decision-making  (depending on the country this could be Bachelor’s degree, Diploma, physiotherapy school, in some countries the profession's entry level is Master’s degree or Professional doctorate) | Education | Hudon et al., 2015; Swisher et al., 2005; Swisher et al., 2002; Trevino, 1996; |
| 26 | 26. My emotions play a role in ethical decision-making | Feelings/emotions | Gillam et al., 2014; Greenfield and Jensen, 2010; Gaudine and Thorne, 2007; McDevitt et al., 2007; |
| 27 | 27. Ethical decision-making requires more skills than just observing a code of conduct or ethical principles | Skills, moral intent(ion) | Greenfield and Jensen, 2010; Swisher et al., 2005; Jones, 1991; |
| 28 | 28. I have the ability to deal with uncertainty in an ethical situation | Skills, ego strength | Hudon et al., 2015; Trevino, 1986; |
| 29 | 29. My personal values play a role in ethical decision-making | Values | Greenfield and Jensen, 2010; |
|  | **Items related to situational, organisational or societal factors** |  | Swisher, 2002; Swisher et al., 2005; Edwards et al., 2011; Jones, 1991; Trevino, 1986; |
| 30 | Contextual factors* of the working system play a role in my ethical decision-making  (characteristics unique to a particular situation, e.g., setting, economic factors, competitive behaviour, resource pressures, institutional context) | Organisational group norms and culture, external pressures, immediate job context, characteristics of the setting | Swisher et al., 2005; Hudon et al., 2015; |
| 31 | Bureaucracy tends to make physiotherapists to follow rules and obligations rather than to think independently about ethical decision-making | Bureaucracy | Greenfield and Jensen, 2010; |
| 32 | Unethical behaviour increases where it stays unpunished | System of reward or punishment, (responsibility for) consequences | Trevino, 1986; Ferrell and Gresham, 1985; |
| 33 | Organisational values have greater influence on my ethical decision-making than my own considerations | Ego strength*, obedience to authority, locus of control*, field dependence* | Hudon et al., 2015; Swisher, 2002;  Trevino, 1986; |
| 34 | A recognisable organisational culture of ethical practice can help a physiotherapist to make a good ethical decision | Organisational group norms and culture | Gaudine and Thorne, 2007; Trevino, 1986; |
| 35 | How senior physiotherapists represent their profession influences the attitude of junior physiotherapists in a good or bad manner | Significant others | Trevino, 1986;  Ferrell and Gresham, 1985; |
| 36 | Physiotherapists need to behave ethically because it is socially expected as their professional role | Code of ethics | Hudon et al., 2015; Greenfield and Jensen, 2010; Edwards et al., 2011; |
| 37 | When ethical behaviour is compromised regularly by a physiotherapists other colleagues will tend to also behave less ethically | Significant others, opportunity (related to the existence or nonexistence of professional codes, system of reward and punishment, and/or corporate policy) | Trevino, 1986;  Ferrell and Gresham, 1985; |
| 38 | Ethical decision-making is more difficult when the factors that contribute to the situation are outside of the realm of my influence | Locus of control*, Moral intensity (characteristics of the issue), field dependence* | Swisher et al., 2005;  Trevino, 1986; |
| 39 | Healthcare system conditions can limit or permit unethical practices | Healthcare system | Swisher et al., 2005; |
| 40 | Contextual factors* can act as barriers or resources to ethical decision-making  (characteristics unique to a particular situation, e.g., geographical location and setting, economic factors, resource pressures, institutional context, payment mechanisms) | Immediate job context, characteristics of the setting, health care system | Swisher, 2002; Swisher et al., 2005; Hudon et al., 2015; |
| 41 | Cultural factors of the country I work in play a role in my ethical decision-making | Cultural and national context | Hudon et al., 2015; Swisher, 2002;  Edwards et al., 2011; |
| 42 | The type/kind* of an ethical situation plays a role in my ethical decision-making  (e.g., an ethical dilemma between competing principles which both seem to be right, a distress of which course of action to take, a temptation of choosing between 'right' and 'wrong' when you would benefit from doing the wrong thing, remaining silent in a situation which appears to be too difficult to be resolved) | Moral intensity (characteristic of the issue) | Swisher et al., 2005; Jones, 1991; |
| 43 | Possible consequences of the decision play a role in my ethical decision-making | (Responsibility for) consequences | McDevitt et al., 2007; Swisher et al., 2005; Jones, 1991; Trevino, 1986; |

*Individual factors that were embedded in the statements in combination with organisational or situational factors.
