# Supplementary material for: Views of physiotherapists on factors that play a role in ethical decision-making: an international online survey study
Source: Arch Physiother. 2023 Feb 1;13:3. doi: 10.1186/s40945-022-00157-y (PMC9889242; doi:10.1186/s40945-022-00157-y)
Supplement: Supplementary file 2 — Additional file 2: Appendix 2. Drop-out analysis by gender, age, nationality (geographic/WP region), and religion. [file 40945_2022_157_MOESM2_ESM.docx]

**Appendix 2. Drop-out analysis by gender, age, nationality (geographic/WP region), and religion.**

|  | **Drop-outs**  **N (% of the total sample)**  **or M (SD)** |
| --- | --- |
| **Gender** |  |
| **Female** | 94 (17%) |
| **Male** | 45 (8.1%) |
| **Diverse** | 1 (0.2%) |
| **Age** |  |
| **N (%)**  **M (SD) yrs.** | 140 (25.27%)  33.69 (10.70) |
| **Geographic/WP region** | |
| **Africa region (AR)** | 10 (1.8%) |
| **Asia Western Pacific region (AWPR)** | 44 (7.9%) |
| **European region (ER)** | 68 (12.2%) |
| **North America Caribbean region (NACR)** | 15 (2.7%) |
| **South America region (SAR)** | 3 (0.6%) |
| **Religion** | |
| **Christianity** | 60 (10.85%) |
| **Islam, Hinduism, Buddhism** | 40 (7.23%) |
| **Secular/Non-religious/Agnostic/**  **Atheist/Irreligious/Unaffiliated** | 22 (3.98%) |
| **Ethnic and indigenous religion, Sikhism Juche, Spiritism, Judaism, Baha’i, Jainism, Neo-Paganism, Unitarian, Don’t want to share this information, other** | 16 (2.89%) |
